# The Treatment of Fractures from a Common-Sense Point of View

**Published:** 1907-10-26

**Authors:** 


### Points in Surgery.

### THE TREATMENT OF FRACTURES FROM A COMMON-SENSE POINT OF VIEW.

### X. FRACTURES OF THE BONES OF THE FOREARM.

Fracture of both bones of the forearm is most often the result of direct violence—indirect violence tends to produce a Colles' fracture. The diagnosis of the injury does not demand more than ordinary skill, for all the characteristic signs and symptoms common to fracture of the long bone of a limb are to be found, if looked for, namely bruising at the site of the injury, unnatural mobility of the limb, loss of function, crepitus and deformity due to displacement. Besides these, a sure indication is supplied by irregularity in the line of the subcutaneous border of the ulna, which is easily detected by the examining finger. When both bones are broken, the fracture is nearly always situated below the insertion of the pronator radii teres; and a good result is easily obtained if the case is properly treated with retentive apparatus. It is not sufficient to apply short splints to the front and back of the forearm and keep it in a sling, as is often taught. If the elbow-joint is not fixed there is a definite risk of delayed union, or even of non-union, just as there is a danger of pseudarthrosis following a fracture of the shaft of the humerus if movement at the shoulder is permitted. It is important to keep the joint immediately proximal to the fracture at rest. In the case of the forearm this is best done by using an internal angular splint, whose upper limb extends to the middle of the humerus and the lower down as far as the palm of the hand. A short posterior splint will be found sufficient. One is advised to insert a small elongated pad along the middle of the flexor aspect of the forearm. The object of this is to press the bones apart, and so prevent the occurrence of vicious union—that is, union of the upper fragment of the radius with the lower fragment of the ulna, or *vice versa.* A moment's thought will serve to show that, however laudable the intention, its employment is quite useless. If it is to perform the services claimed for it, the splints would have to be bandaged to the arm so tightly that the patient could not stand the discomfort, and there would be no danger of a subsequent Volckmann's contracture. What generally happens is that the pad is put in as a routine measure, and serves no serious purpose at all, except to make the patient uncomfortable. A more certain and a more scientific way of avoiding vicious union is to employ passive movements at as early a date as possible. The forearm should be gently pronated and supinated daily from about the end of the first week until union is fairly firm.

#### Fractures of the Radius only.

The radius may be fractured at any point in its shaft, but for clinical purposes such fractures are divided into two groups: (1) fracture above the insertion of the pronator radii teres, and (2) fracture below that level. In the first group, the biceps cubiti and the pronator radii teres, which are antagonistic muscles, can both exert their full action unopposed. It follows that the upper fragment is fully supinated by the former and the lower is pronated by the latter muscle. The upper fragment is small, and is well covered by muscles, so that the surgeon has no control over it. The lower fragment, on the other hand, can be moved into, and kept in, any position. In order to bring the fragments into apposition, it is necessary therefore to supinate the lower part fully, and fix the arm on a posterior angular splint. The position is somewhat irksome, but the patient soon gets accustomed to it; and the result obtained is usually quite good. When the radius is fractured below the insertion of the pronator radii teres, the upper fragment is maintained in a position half way between pronation and supination, because the supinating action of the biceps is antagonised by the pronator radii teres. In this case the lower fragment should be manipulated into line with the upper, and retained there with an anterior and posterior splint, as in fracture of both bones. As in the latter injury, the elbow should be fixed.

#### Colles' Fracture.

This is a transverse fracture of the lower end of the radius, as the result of indirect violence, such as falling on the outstretched hand. It is a common accident, especially in alcoholic old women. The displacement is most characteristic. The lower fragment is pushed backwards in the direction of the force producing the injury, and is rotated round a transverse axis, so that the articular surface looks backwards as well as downwards. The whole hand is abducted towards the radial side. The upper fragment is pronated and approximated to the ulna. Certain abnormal prominences can be detected due to the ulnar styloid process on the inner side, to the lower end of the upper fragment on the flexor aspect, and to the lower fragment on the extensor. Impaction occurs fairly frequently. The only condition for which it can possibly be mistaken is separation of the lower epiphysis of the radius, which, unlike Colles' fracture, occurs in young subjects, and is usually associated with a similar condition in the ulna.

The successful treatment of a Colles' fracture is not at all a difficult matter. The deformity is easily reduced under gas by extension and counter-extension, and there is no tendency for it to recur because it is kept in position by the tendons surrounding the wrist-joint. The best splint to use is Carr's, which was specially invented for this injury. It consists of a straight wooden splint, to whose lower end is attached a rounded piece of wood about four inches long and three-quarters of an inch in diameter. This fits into the palm, and is set at an angle, so as to reduce the radial abduction of the hand. Above this is a shallow depression, to receive the protuberance caused by the thenar and hypothenar eminences. Unlike other splints it is not padded, but simply has a covering of lint. This splint is fixed by bandages to the forearm and hand, which is carried in a sling. Early movement is essential, lest stiffness due to tenosynovitis or to adhesions between the tendon sheaths and the bone should supervene. The fingers therefore should be moved daily from the beginning: after a week or ten days the wrist may be gently moved as well, and the splint may be dispensed with altogether after three weeks.

